# Neuroplastic effects of end-effector robotic gait training for hemiparetic stroke: a randomised controlled trial

**DOI:** 10.1038/s41598-020-69367-3

**Published:** 2020-07-27

**Authors:** Hayeon Kim, Gyulee Park, Joon-Ho Shin, Joshua H. You

**Affiliations:** 1 Translational Research Center for Rehabilitation Robots, National Rehabilitation Center, Seoul, Republic of Korea; 2 Department of Rehabilitation Medicine, National Rehabilitation Center, 58, Samgaksan-ro, Gangbuk-gu, Seoul, 01022 Republic of Korea; 3 Department of Physical Therapy, Dynamic Movement Institute and Technology, College of Health Science, Sports Movement Artificial-Intelligence Robotics Technology (SMART) Institute, “Yonsei GOODWELLNESS Center” for Sports, Wellness, and Fitness Across Life Span Disabilities, Yonsei University, 1 Yonseidae-gil, Wonju, Gangwon-do 26493 Republic of Korea

**Keywords:** Neuroscience, Health care, Medical research

## Abstract

Detecting neuroplastic changes during locomotor neurorehabilitation is crucial for independent primal motor behaviours. However, long-term locomotor training-related neuroplasticity remains unexplored. We compared the effects of end-effector robot-assisted gait training (E-RAGT) and bodyweight-supported treadmill training (BWST) on cortical activation in individuals with hemiparetic stroke. Twenty-three men and five women aged 53.2 ± 11.2 years were recruited and randomly assigned to participate in E-RAGT (n = 14) or BWST (n = 14) for 30 min/day, 5 days/week, for 4 weeks. Cortical activity, lower limb motor function, and gait speed were evaluated before and after training. Activation of the primary sensorimotor cortex, supplementary motor area, and premotor cortex in the affected hemisphere significantly increased only in the E-RAGT group, although there were no significant between-group differences. Clinical outcomes, including the Fugl-Meyer assessment (FMA), timed up and go test, and 10-m walk test scores, improved after training in both groups, with significantly better FMA scores in the E-RAGT group than in the BWST group. These findings suggest that E-RAGT effectively improves neuroplastic outcomes in hemiparetic stroke, although its superiority over conventional training remains unclear. This may have clinical implications and provides insight for clinicians interested in locomotor neurorehabilitation after hemiparetic stroke.

Trial Registration: ClinicalTrials.gov Identifier NCT04054739 (12/08/2019).

## Introduction

End-effector robot-assisted gait training (RAGT) in combination with exoskeleton RAGT has recently gained clinical acceptance as a tool for improving clinical and biomechanical parameters^1,2^, as well as improving the associated neuroplastic changes^3^ in locomotor control, in individuals with hemiparetic stroke. Conventionally, bodyweight-supported treadmill training (BWST), which is conceptually based on the task-specific model, has been used to induce functional, biomechanical, and neuroplastic changes during the locomotor rehabilitation of adults with hemiparetic stroke. Clinical studies have shown that BWST improves Fugl-Meyer assessment (FMA) scores and gait speed to a degree comparable with conventional physical therapy in stroke patients^4,5^. According to the latest Cochrane review, BWST did not improve the chances of gait independently compared with conventional physical therapy; however, BWST increased gait velocity and endurance significantly compared with conventional physical therapy^6^.

Despite the potential clinical and neuromechanical benefits provided by BWST, a minimum of two physical therapists are required to guide the legs and hips of the patient walking on a treadmill equipped with a built-in overhead harness^7,8^. To mitigate the labour intensiveness of the procedure, various robotic locomotor systems have been devised to automate and enhance locomotor training. Furthermore, recent studies in neuroscience and robotic translational research have demonstrated the importance of therapy dosage and intensity, high repetitiveness, and task-oriented paradigms. These insights led to the development of end-effector robot-assisted gait training (E-RAGT), which was designed to improve insufficient inter-limb ankle-knee-hip coordination during hemiparetic gait. The hemiparetic gait cycle is characterised by a lack of ankle dorsiflexion kinematics during the initial contact and swing phases, and insufficient plantarflexion moment leading from the terminal stance into the pre-swing phase, resulting in uncoordinated ankle-knee-hip inter-limb movements^9,10^. The end-effector robot operates with the patient’s feet strapped to independent foot plates moving along programmable gait trajectories for the vertical and horizontal components of the centre of mass, and it provides the patient with guidance and real-time visual feedback. The main difference between E-RAGT and exoskeleton RAGT is that E-RAGT utilizes foot plates with an end-effector device attached to a double crank and a rocker gear system to permit ankle dorsiflexion and plantar flexion movement during gait training; this provides less control over the hip and knee joints to allow active movement. In contrast, the exoskeleton RAGT consists of either an actuated hip-knee joint or a hip-knee-ankle joint, both of which provide an accurate control of inter-joint coordination but allow less active movement during gait training. Clinical RAGT studies have shown improvements in independent gait scale, lower limb motor function, and walking speed^11,12^. Such functional locomotor recovery has been associated with neuroplastic improvement in hemiplegic stroke, including increased motor-evoked potential amplitude in repetitive transcranial magnetic stimulation studies^13–15^; restoration of the corticospinal tract in diffusion tensor tractography studies^16,17^; bihemispheric reorganisation evolved from contralesional primary sensorimotor cortex (SMC) to ipsilesional SMC in functional magnetic resonance imaging (fMRI) studies^18–20^; and dissolution of abnormal activations and increased activation of the ipsilesional SMC in an fMRI study^21^. However, this hypothesis has not been tested with E-RAGT to date.

Neuroplastic change during locomotor neurorehabilitation is universally recognised as a crucial factor for the recovery of independent primal motor behaviours. Comparative functional near-infrared spectroscopy (fNIRS) neuroimaging studies have provided insights into the neural substrates of the locomotor neural network and their functional roles. Clinical neuroscientists have employed a sophisticated fNIRS neuroimaging technique to measure blood oxygenation changes during treadmill walking, and have demonstrated a bilateral increase in oxygenated haemoglobin (oxyHb) in the SMC and supplementary motor area (SMA) in adults with^22^ and without hemiparetic stroke^23^. Similarly, a recent fNIRS study in healthy participants showed increased SMC, SMA, and premotor cortex (PMC) activation during exoskeleton RAGT compared with treadmill walking or stepping^3^. However, the long-term effects of conventional treadmill-based gait training and E-RAGT on cortical activation and locomotor control mechanisms in adults with hemiparetic stroke remain unknown. In the current fNIRS literature, the long-term locomotor training-related effects on neuroplastic mechanisms and the roles of the underlying neural substrates have not been fully explored.

Thus, the purpose of this study was to compare, in individuals with hemiparetic stroke, the effects of E-RAGT vs. BWST on cortical activation and clinical outcomes, including lower limb motor function and gait speed. We hypothesised that E-RAGT would have superior effects on both cortical activation and clinical outcomes compared to BWST.

## Methods

**Figure 1:**
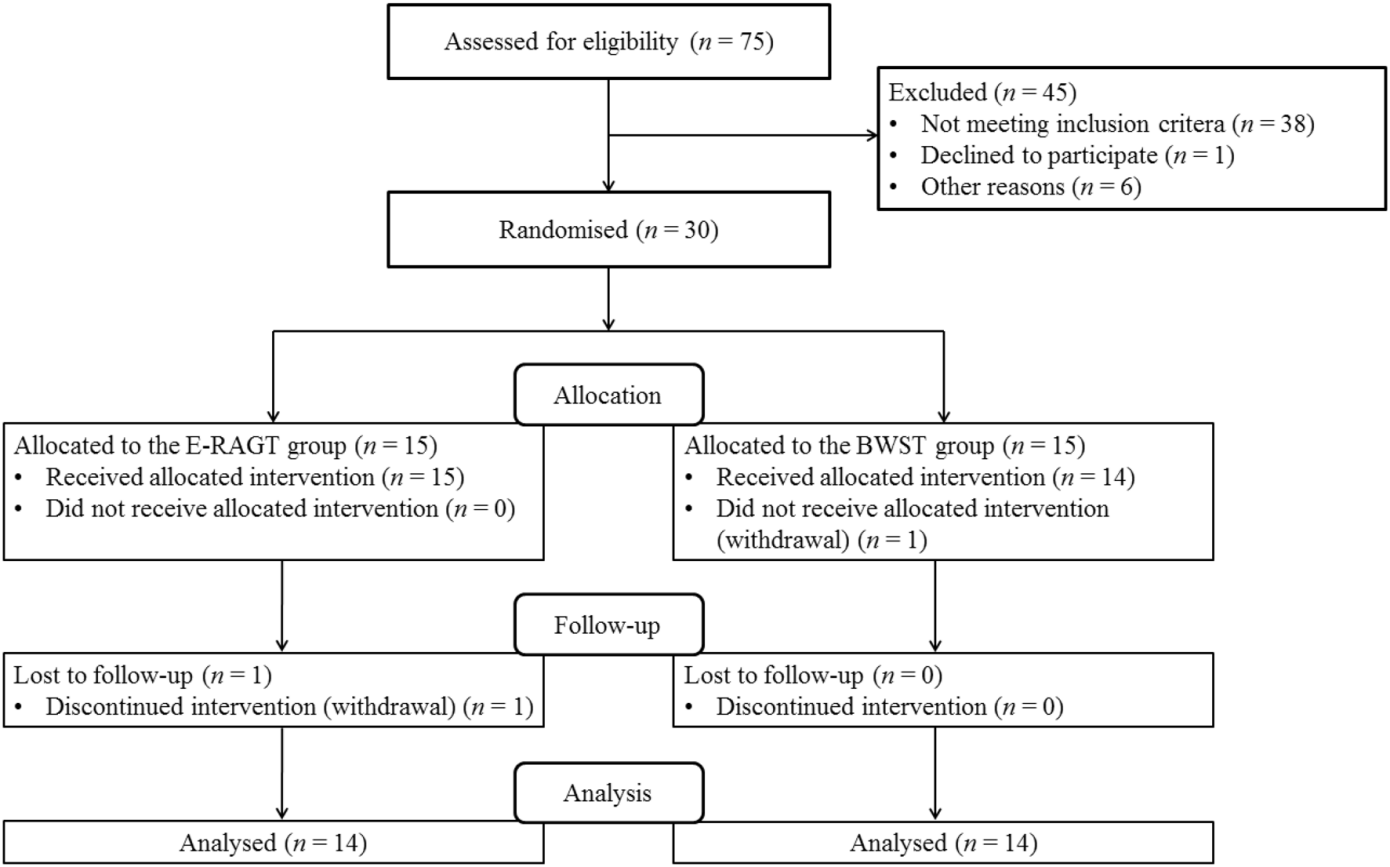
Flow diagram for the study. *E-RAGT* end-effector robot-assisted gait training, *BWST* bodyweight-supported treadmill training.

Thirty volunteer participants with hemiparetic stroke (25 men, 5 women; mean age ± standard deviation, 54 ± 11 years) were recruited from inpatients of the National Rehabilitation Center, Seoul, Republic of Korea. After the subjects were recruited via bulletin board notices within the hospital, initial screening was conducted to determine whether the potential participants met the inclusion criteria. The inclusion criteria of this study were (1) hemiplegia due to a first-ever stroke; (2) time elapsed after stroke onset, 3–12 months; (3) supervision-dependent ambulation (functional ambulation category level = 3); and (4) Korean Mini-Mental State Examination score > 24. The exclusion criteria were: (1) orthopaedic problems or muscle diseases which cause impairments in mobility, (2) high risk of spontaneous fracture (assessed using the Computerized Bone Mineralometry score), and (3) other neurological diseases (Table 1). The experimental protocol was approved by the Ethical Committee of the Korea National Rehabilitation Center (Institutional Review Board approval number: NRC-2017-02-015). All participants received explanations about the experiment before its start and voluntarily agreed to participate. Written informed consent was provided by all participants. The participants were randomly allocated to either the E-RAGT or the BWST group using a randomisation table with a 1:1 allocation ratio. A researcher generated the random allocation sequence, another researcher assigned participants to interventions, and a third-party blinded researcher assessed outcome measures. The randomisation assignments were concealed in consecutively numbered, sealed, opaque envelopes. The study was conducted in accordance with the Declaration of Helsinki. A flow diagram for the study is presented in Fig. 1. No subjects experienced adverse events, but two subjects, one per group, dropped out due to sudden discharge and personal reasons. Table 2 summarises the demographic and clinical characteristics of the participants. There were eight cases of left hemiplegia in the E-RAGT group and four cases in the BWST group. Most studies have reported visuo-spatial neglect is more frequent and severe in left hemiplegia rather than right hemiplegia^24^. To prevent this potential bias, patients with severe neglect were excluded.

**Table 1:** Inclusion and exclusion criteria.

Inclusion criteria	Exclusion criteria
Hemiplegia due to a first-ever stroke Time elapsed after stroke onset, 3–12 months Supervision-dependent ambulation (functional ambulation category level = 3) Korean Mini-Mental State Examination score > 24	Orthopaedic problems or muscle diseases that cause impairments in mobility High risk of spontaneous fracture (assessed using the Computerized Bone Mineralometry score) Other neurological diseases

**Table 2:** Demographic and clinical characteristics of the participants.

	E-RAGT (*n* = 14)	BWST (*n* = 14)	*p*
Sex (male/female)	11/3^a^	12/2	1.000*
Age	55 ± 12^b^	51 ± 10	0.430^†^
Height (cm)	170.29 ± 9.15	168.71 ± 7.71	0.627^†^
Weight (kg)	67.14 ± 9.62	67.43 ± 8.90	0.936^†^
Post-stroke time (mon)	6.14 ± 2.83	5.79 ± 2.67	0.734^†^
Type (haemorrhage/infarct)	6/8	5/9	1.000*
Paretic side (right/left)	6/8	10/4	0.252*
Brunnstrom recovery stage	3.36 ± 1.21	2.93 ± 1.07	0.332^†^
FAC	3.00 ± 0.00	3.00 ± 0.00	1.000^†^
BBS	44.21 ± 3.72	44.14 ± 4.99	0.966^†^

*E-RAGT*, end-effector robot-assisted gait training, *BWST* bodyweight-supported treadmill training, *FAC* functional ambulation category, *BBS* Berg Balance Scale.

*χ^2^ test, ^†^*t*-test.

^a^Number, ^b^Mean ± standard deviation.

**Figure 2:**
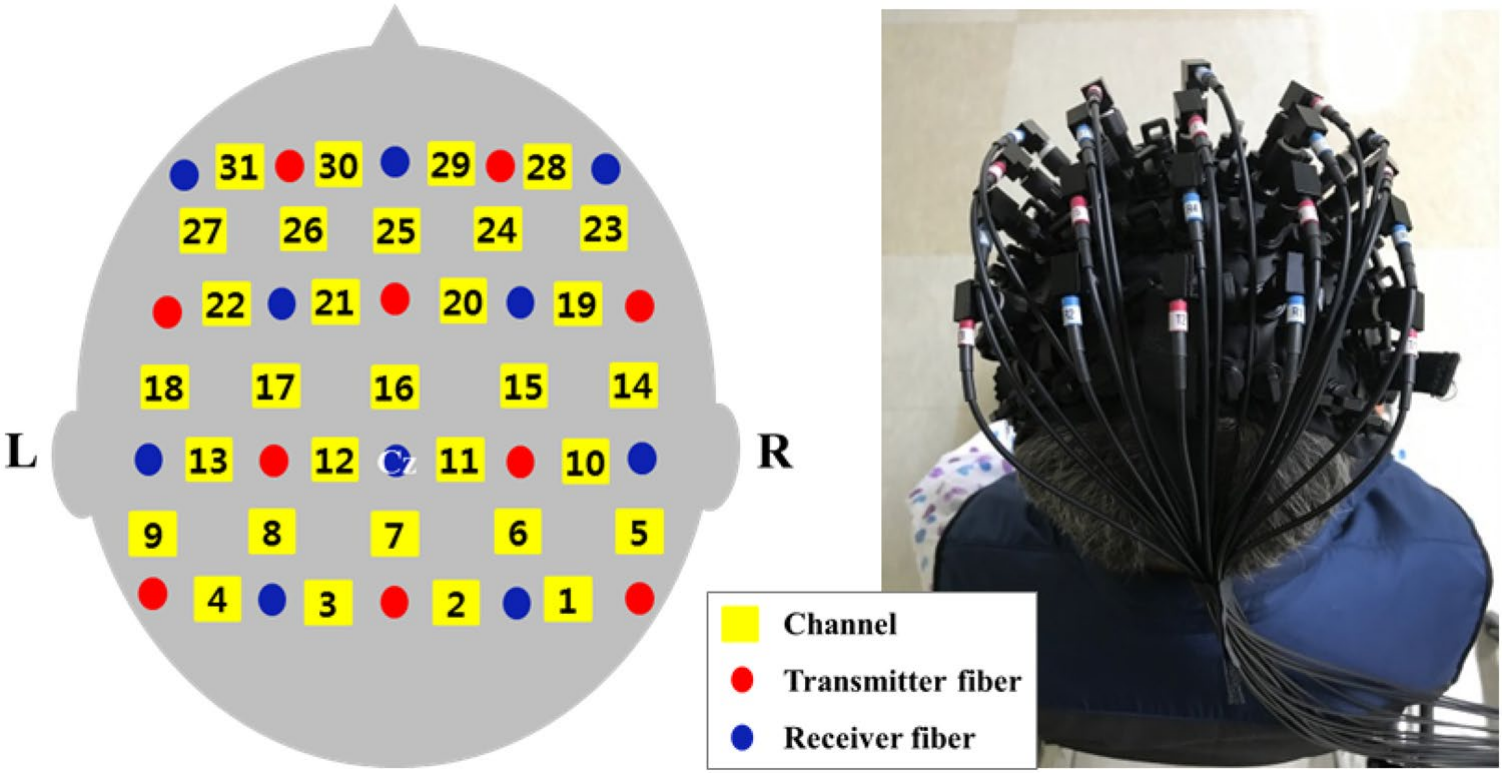
The placement of channels and probes for functional near-infrared spectroscopy.

A fNIRS optical imaging system (LABNIRS; Shimadzu, Kyoto, Japan) was used to record cortical activity-related changes in oxyHb with continuous-wave, laser-diode wavelengths of 780, 805, and 830 nm. The fNIRS system consists of 20 optodes with 10 transmitter and 10 receiver fibres, allowing the simultaneous recording of 31 channels. Each optode was superimposed on the whole brain image, with channel numbers from 1 to 31 assigned. Measurements were made at a sampling rate of 30 Hz. The optical imaging data were normalised to a standard stereotaxic space, the Montreal Neurological Institute brain template, using the software package NIRS-Statistical Parametric Mapping (NIRS-SPM) implemented in the MATLAB environment (MathWorks, Natick, MA, USA). The international 10–20 system was used to identify the locations of the optodes, with the Cz (cranial vertex) located beneath the fourth receiver fibre, between the 11th and 12th channels (Fig. 2). The optodes were secured at an inter-optode distance of 3.0 cm on the skull using a holder cap made of thermoplastic resin. The oxyHb changes associated with cortical activity were then recorded in predetermined regions of interest (12 × 9 cm), including the SMC, SMA, PMC, pre-supplementary motor (pre-SMA), and prefrontal cortex (PFC). Each fNIRS experiment included three repetitions of a rest-task-rest (30–60–30 s) block design. The task consisted of overground walking along a walkway at a self-selected gait speed. During the rest period, participants were instructed to stand in a relaxed position with their arms beside the trunk, without performing any movements. The participants were instructed to stand for 30 s (rest period), walk for 60 s in response to a cue sound that marked the beginning of the task period, and stop walking and stand still in response to a second cue sound that marked the end of the walking period. This process was repeated three times. The middle 30 s of each task period were used for the analysis. Secondary outcome measures included the lower-extremity subscale of FMA, the timed up and go test (TUG), and 10-m walk test (10MWT). Clinical evaluations were performed by one skilled physical therapist. The cortical activity-related changes were assessed at baseline (pre-test) and after 4 weeks of the intervention (post-test). The FMA, TUG, and 10MWT were assessed at baseline (pre-test), after 2 weeks of the intervention (mid-test), and after 4 weeks of the intervention (post-test).

The E-RAGT group received end-effector-based RAGT and the BWST group received treadmill gait training with partial bodyweight support 30 min/day, 5 times a week, for 4 weeks, for a total of 20 sessions^25,26^. E-RAGT was performed using the G-EO System Evolution (Reha Technology, Olten, Switzerland). The harness secured to the participants on two foot plates, whose trajectories could be programmed to approximate a normal gait. During training, the participants received real-time visual feedback from the pressure plates regarding the weight distribution on their feet. They were also provided with verbal cues to help them ensure that their pelvis and trunk were centred and their movements were symmetrical. The specific setting parameters including bodyweight support and step length were adjusted based on the individual’s weight and height. The E-RAGT initially began with 30% bodyweight support and a speed of 0.8 km/h to allow for adaptation to the robotic device^25,26^. To assist participants in focusing on the timing of the gait pattern with moderate intensity, the amount of bodyweight supported was gradually reduced to 0%, and the speed gradually increased to 2.0 km/h, participant ability permitting. The first week was dedicated to passive-mode training. This was then changed to an active-assistive mode, participant ability permitting, by gradually reducing the amount of guidance. Passive mode served to help patients track the predefined trajectories and recover movement ability. Active-assistive mode served to encourage patients to trigger robotic assistance by their own active efforts. In this mode, the robot provided assistance when the participant showed some voluntary impulse to move by applying a force to the footplates above a selected threshold. The threshold value was gradually increased according to participant progress.

In the BWST group, participants wore a waist harness to ensure safety and body-weight support during training. The BWST began with 30% bodyweight support and a speed of 0.8 km/h. To assist participants in focusing on the timing of the gait pattern with moderate intensity, the bodyweight support was gradually reduced to 0% and speed gradually increased to 2.0 km/h. During this training, a physical therapist manually assisted hemiparetic lower limb movement if needed.

### Statistical analysis

All data were analysed using SPSS for Windows, version 20.0 (IBM Corp., Armonk, NY, USA). The Kolmogorov–Smirnov test was used to confirm that all continuous variables were normally distributed. To evaluate our randomisation procedure, the baseline differences of all variables between the E-RAGT and BWST groups were analysed using the independent *t*-test or the χ^2^ test. Paired *t*-tests were used to compare pre-test and post-test cortical activity variables in each group. A two-way mixed repeated-measures analysis of variance (ANOVA) with factors of group (E-RAGT and BWST) and times (pre-test and post-test) was used to determine the Group × Time interaction for cortical activity variables. Repeated-measures ANOVA was used to compare pre-test, mid-test, and post-test clinical measurements in each group. Post-hoc analysis using the Bonferroni analysis was done for the variables which showed a statistically significant difference. A two-way mixed repeated-measures ANOVA with factors of group (E-RAGT and BWST) and times (pre-test, mid-test, and post-test) was used to assess the Group × Time interaction for clinical measurements variables. Results are expressed as means ± standard deviations. The significance level was set at *p* < 0.05.

## Results

### Primary outcome measures

Table 3 presents the regional cortical activities of the E-RAGT and BWST groups during the overground gait test before and after intervention. No significant Group × Time interaction in cortical activity was noted in any region. The cortical activity of the SMC, SMA, and PMC on the affected hemisphere showed no significant Group × Time interaction (F = 0.112, 0.024, 0.106; *p* = 0.741, 0.878, 0.747; partial η^2^ = 0.004, 0.001, 0.004, respectively) and no significant main effects for any group (F = 0.177, 2.594, 0.760; *p* = 0.677, 0.119, 0.391; partial η^2^ = 0.007, 0.091, 0.028, respectively), however, significant main effects were observed for time (F = 4.864, 9.294, 6.601; *p* = 0.036, 0.005, 0.016; partial η^2^ = 0.158, 0.263, 0.202, respectively). In the E-RAGT group, significant within-group differences between pre-test and post-test were noted for the activity of the SMC (*p* = 0.016), SMA (*p* = 0.022), and PMC (*p* = 0.009) over the affected hemisphere. However, in the BWST group, no significant differences were observed in any region between pre-test and post-test.

**Table 3:** Regional cortical activity (mM·cm) during gait in the E-RAGT and BWST groups.

Group	Pre-test	Post-test	Within-group *p*-value	ANOVA *p*-value		
				Group	Time	Interaction
**AH SMC**						
E-RAGT	0.00494 ± 0.00257^a^	0.00912 ± 0.00205	0.016*	0.677	0.036	0.741
BWST	0.00714 ± 0.00280	0.01022 ± 0.00421	0.470			
**UH SMC**						
E-RAGT	0.00580 ± 0.00335	0.00798 ± 0.00207	0.363	0.405	0.709	0.566
BWST	0.01085 ± 0.00365	0.01038 ± 0.00449	0.730			
**AH SMA**						
E-RAGT	0.00574 ± 0.00387	0.01075 ± 0.00370	0.022*	0.119	0.005	0.878
BWST	0.00041 ± 0.00176	0.00410 ± 0.00217	0.124			
**UH SMA**						
E-RAGT	0.00796 ± 0.00369	0.00880 ± 0.00188	0.397	0.050	0.575	0.817
BWST	0.00066 ± 0.00196	0.00266 ± 0.00359	0.551			
**AH PMC**						
E-RAGT	0.00340 ± 0.00236	0.00913 ± 0.00228	0.009*	0.391	0.016	0.747
BWST	0.00139 ± 0.00175	0.00583 ± 0.00355	0.363			
**UH PMC**						
E-RAGT	0.00820 ± 0.00282	0.00838 ± 0.00209	0.683	0.474	0.323	0.380
BWST	0.00451 ± 0.00182	0.00735 ± 0.00315	0.245			
**AH pre-SMA**						
E-RAGT	0.00395 ± 0.00338	0.00887 ± 0.00201	0.109	0.879	0.092	0.619
BWST	0.00566 ± 0.00305	0.00838 ± 0.00407	0.331			
**UH pre-SMA**						
E-RAGT	0.00229 ± 0.00335	0.00614 ± 0.00226	0.177	0.484	0.404	0.567
BWST	0.00775 ± 0.00411	0.00848 ± 0.00645	0.551			
**AH PFC**						
E-RAGT	0.00575 ± 0.00223	0.00721 ± 0.00170	0.331	0.312	0.536	0.819
BWST	0.00327 ± 0.00192	0.00395 ± 0.00311	0.975			
**UH PFC**						
E-RAGT	0.00629 ± 0.00239	0.00680 ± 0.00283	0.730	0.948	0.731	0.946
BWST	0.00593 ± 0.00234	0.00670 ± 0.00356	0.925			

At baseline, there were no significant differences between the E-RAGT and BWST groups for any variable.

*E-RAGT* end-effector robot-assisted gait training, *BWST* bodyweight-supported treadmill training, *AH* affected hemisphere, *UH* unaffected hemisphere, *SMC* primary sensorimotor cortex, *SMA* supplementary motor area, *PMC* premotor cortex, *pre-SMA* pre-supplementary motor area, *PFC* prefrontal cortex.

*Significant within-group difference, based on paired *t*-test, *p* < 0.05.

^a^Mean ± standard deviation.

### Secondary outcome measures

Table 4 presents the clinical measurements of the E-RAGT and BWST groups at three time points: pre-test, mid-test, and post-test. For FMA values, there was a significant Group × Time interaction (F = 4.892; *p* = 0.011; partial η^2^ = 0.158). However, there was no significant effect of Group × Time in the TUG or 10MWT results. In the E-RAGT group, significant within-group differences between pre-test and post-test were noted for FMA (*p* < 0.001), TUG (*p* = 0.001), and 10MWT (*p* = 0.024). In the BWST group, the FMA (*p* = 0.013), TUG (*p* = 0.004), and 10MWT (*p* = 0.019) scores significantly improved after intervention.

**Table 4:** Clinical measurements in the E-RAGT and BWST groups.

Group	Pre-test	Mid-test	Post-test	Within-group *p*-value	Post-hoc *p*-value			ANOVA *p*-value		
					Pre vs Mid	Mid vs Post	Pre vs Post	Group	Time	Interaction
**FMA**										
E-RAGT	21.93 ± 3.43^a^	23.00 ± 2.66	24.79 ± 3.42	< 0.001*	0.026	0.005	< 0.001	0.759	< 0.001	0.011^†^
BWST	23.21 ± 2.55	23.29 ± 2.43	24.21 ± 3.26	0.047*	0.720	0.042	0.013			
**TUG (s)**										
E-RAGT	43.90 ± 19.13	38.30 ± 16.85	35.18 ± 16.35	0.002*	0.015	0.248	0.001	0.651	< 0.001	0.914
BWST	40.43 ± 19.77	36.12 ± 17.26	32.14 ± 14.45*	0.004*	0.108	0.037	0.004			
**10MWT (m/s)**										
E-RAGT	0.32 ± 0.20	0.35 ± 0.18	0.35 ± 0.17	0.012*	0.034	0.991	0.024	0.857	0.001	0.162
BWST	0.32 ± 0.15	0.35 ± 0.17	0.40 ± 0.21	0.013*	0.149	0.060	0.019			

At baseline, there were no significant differences between the E-RAGT and BWST groups for any variable.

*E-RAGT* end-effector robot-assisted gait training, *BWST* bodyweight-supported treadmill training, *FMA* Fugl–Meyer assessment, *TUG* timed up and go test, *10MWT* 10-m walk test.

*Significant within-group difference, based on repeated-measures analysis of variance (ANOVA), *p* < 0.05.

^†^Significant Group × Time interaction effect, based on two-way mixed repeated-measures ANOVA with factors of group (E-RAGT and BWST) and times (pre-test, mid-test, and post-test), *p* < 0.05.

^a^Mean ± standard deviation.

## Discussion

The present investigation is the first randomised clinical trial to emphasise the comparative effects of long-term E-RAGT and BWST interventions on cortical activity and gait function in individuals with hemiparetic stroke. We found no significant difference between the E-RAGT and BWST groups for cortical activity in any region. However, the E-RAGT intervention led to significant improvements in SMC, SMA, and PMC activity on the affected hemisphere between pre-test and post-test. In contrast, the BWST intervention showed no significant difference in cortical activity between pre-test and post-test. Clinical outcomes, including FMA, TUG, and 10MWT scores, were improved after intervention in both groups, but only the FMA score showed a significantly greater improvement in the E-RAGT group than in the BWST group. Most importantly, the fNIRS data demonstrated neuroplastic changes in the SMC, SMA, and PMC of the affected hemisphere, but only for the pre-test vs. post-test comparison in the E-RAGT group. No previous evidence of neural plasticity induced by robotic locomotor training exists. It is therefore difficult to compare our fNIRS data with previous data on stroke.

The fNIRS data analysis revealed no substantial differences in cortical oxyHb changes between groups. However, cortical oxyHb levels significantly increased only in the robotic locomotor training group, and only after 4 weeks of training. After the E-RAGT intervention, cortical activity, as demonstrated by oxyHb levels, was greater in the SMC (84.62%), SMA (87.28%), and PMC (173.82%) in the affected hemisphere. Certainly, our novel fNIRS findings support the hypothesis that robotic locomotor-training induced cortical reorganisation. This finding is consistent with previous neuroimaging evidence, which demonstrated increased activation coherence between the cortical electroencephalogram (primary motor cortex) and the tibialis anterior electromyogram in healthy participants during treadmill gait^27^. A longitudinal fNIRS study also revealed a substantial increase in cortical oxyHb levels in the ipsilesional SMC and PMC during locomotor activity, together with an enhancement of locomotor function, after 2 months of rehabilitation of inpatients with subacute stroke^28^. Another fNIRS cross-sectional study of healthy participants revealed more activation in the SMC-PMC-SMA motor control network during robotic gait than stepping gait or treadmill gait^3^. The factors possibly underlying robotic training-induced neuroplasticity are real-time, accurate kinematic feedback and visual feedback of weight-distribution information. Correct visual and proprioceptive feedback are important sensory inputs in cortical motor learning^29^ that help to predict and adjust locomotor outcomes^30,31^.

Interestingly, a previous fMRI study showed global, bilateral SMC activation before virtual reality intervention in patients with chronic hemiparetic stroke; however, after the intervention, the SMC activation shifted from the unaffected to the affected hemisphere and became more localised; this shift was correlated with enhanced locomotor recovery^20^. A similar fMRI study^32^ investigated walking ability and cortical reorganisation after 4 weeks of locomotor training using a treadmill with partial bodyweight support in patients with chronic stroke. The study demonstrated that the involvement of bilateral SMC activation is crucial in improving a complex behaviour, such as walking, despite the strong subcortical contributions to gait control.

Contemporary neuroscientific evidence suggests that normal automatic locomotor rhythm and pattern is regulated by central pattern generators (CPGs) in the spinal cord, subsequently modified by peripheral sensory inputs, and mediated under supraspinal control^22,33,34^. Specifically, these CPGs receive modulation from supraspinal locomotor centres, including the subthalamic locomotor region in the lateral hypothalamic area; the mesencephalic locomotor region, corresponding to the cuneiform and pedunculopontine nuclei in the dorsal midbrain; the cerebellar locomotor region, located close to the fastigial nuclei in the cerebellar midline; and the pontine locomotor region in the pontomedullary reticular formation^35–37^. However, perilesional or contralesional cortical modulation may be involved in the locomotor control process in adults with hemiparetic stroke because the corticospinal motor pathways regulating the supraspinal locomotor centres are interrupted in such cases^38^. The corticoreticulospinal tracts innervate whole spinal segments, which control postural muscle tone, symmetric postural sets, and anticipatory postural adjustment, preceding gait initiation^39,40^. When these corticoreticulospinal pathways are affected, asymmetrical postural tone and impaired postural adjustment become evident during gait^41^. Accumulating evidence suggests that locomotor recovery in hemiparetic stroke involves a cortical activity shift, whereby the contralesional motor area is first activated in compensation prior to any intervention and then the ipsilesional motor area becomes more active after the intervention^42–46^. Therefore, the improvement in activity of the SMC in the affected hemisphere observed in our study may be meaningful for stroke recovery. A recent fMRI study^47^ that investigated brain activation during robotic step-like walking demonstrated an increase the activity of the SMC (as well as the intraparietal sulcus and superior parietal cortex) and subcortical and cerebellar regions, further confirming the importance of the cortical role in initiation and termination, and the subcortical in regulation, of locomotion^41^. Moreover, in hemiparetic stroke, postural and locomotor functions are impaired; hence, the cortex becomes more involved in postural responses to alterations in cognitive state, initial sensory-motor conditions, and prior perturbation experience, all of which influence changes in the ‘central set’^48^. The cortex regulates the central set for postural responses via the cerebellar-cortical loop by adapting postural responses based on prior experience^49–51^, whereas the basal ganglia-cortical loop exerts control by pre-selecting and optimising postural responses based on the current context^52,53^. The cerebral cortex may control postural responses both directly, through long-latency corticospinal loops, and indirectly, through shorter-latency postural responses produced by modulating the supraspinal centres (midbrain and brainstem) that activate the synergistic postural muscles during locomotion. The locomotor neuronal signals are transmitted to the brainstem via the corticoreticulospinal pathways, which subcortically regulate anticipatory postural sets during locomotion. In coordination with supraspinal regulation, the corticospinal system generates accurate foot placement and limb kinematic trajectory, which are required for locomotor tasks^41^. In particular, the PMC and SMA are involved in purposeful adjustment and control during locomotion through connections with the basal ganglia, brain stem, cerebellum, and spinal cord^35,54,55^; our finding of improved PMC and SMA activity might be related to enhanced control of locomotor function.

In this study, E-RAGT was more effective than BWST in stroke patients only in terms of FMA scores. The lower limb motor function in locomotion may have contributed to the locomotor retraining paradigm, which highlighted gradually rhythmic, repetitive, and concentrated practice. Previous studies of locomotor learning showed that traditional gait training in patients with stroke, which provided approximately 292 steps per session, may be insufficient to improve function and neuroplasticity^56^. It has been suggested that at least 300–500 repetitions are required for the recovery of lower limb motor function and neuroplasticity in patients with stroke^57,58^. This may be the result of the repetitive exercise of walking more precisely assisted by the robot during E-RAGT, as opposed to treadmill training. The BWST also provided repetitive motor relearning, but it may be insufficient for delivering accurate sensory input compared with E-RAGT, even with the physical therapist’s manual assistance.

The present study has several limitations that could be addressed in future studies. First, the sample size of the two groups was relatively small, and this might have hindered the detection of some differences in the outcomes between the two groups; thus, a study with a large patient group is recommended. Second, fNIRS can only measure cortical (not subcortical) activity. Since the locomotor behaviour of healthy adults is believed to be mediated by CPGs and supraspinal modulation, future studies are required to measure subcortical as well as cortical activity to better understand the neuroplasticity of locomotor training. Third, the intervention might have been of insufficient intensity and duration to produce noticeable neuroplastic change. Further studies are needed to confirm neuroplastic change by performing higher intensity and longer duration interventions.

In conclusion, the present study provides the first empirical evidence of the neuroplastic effects of E-RAGT in individuals with hemiparetic stroke. No substantial differences in baseline cortical activity were found between the E-RAGT and BWST groups; however, the oxyHb levels of the SMC, SMA, and PMC in the affected hemisphere were significantly increased only after 4 weeks of E-RAGT. Clinical outcomes, including FMA, TUG, and 10MWT, improved after 4 weeks of training in both the E-RAGT and BWST groups. Our study showed that E-RAGT led to significantly improved FMA scores compared with BWST; however, this was the only significant clinical difference between the two treatments. Therefore, the results suggest that E-RAGT is effective for improving neuroplastic and clinical outcomes in individuals with hemiparetic stroke, although any superiority it may have over conventional training was not confirmed. Our findings may have clinical implications, and provide insight to clinicians interested in locomotor neurorehabilitation in individuals with hemiparetic stroke.

## Data Availability

The datasets generated during and analysed during the current study are available from the corresponding author on reasonable request.
